# Modulation of renal oxygenation and perfusion in rat kidney monitored by quantitative diffusion and blood oxygen level dependent magnetic resonance imaging on a clinical 1.5T platform

**DOI:** 10.1186/s12882-016-0356-x

**Published:** 2016-10-03

**Authors:** Neil P. Jerome, Jessica K. R. Boult, Matthew R. Orton, James d’Arcy, David J. Collins, Martin O. Leach, Dow-Mu Koh, Simon P. Robinson

**Affiliations:** 1 Cancer Research UK Cancer Imaging Centre, Division of Radiotherapy & Imaging, The Institute of Cancer Research, London, SM2 5NG UK; 2 Department of Radiology, Royal Marsden NHS Foundation Trust, Sutton, Surrey SM2 5PT UK

**Keywords:** Diffusion, BOLD, Renal, Imaging, Furosemide, Angiotensin, Hydralazine

## Abstract

**Background:**

To investigate the combined use of intravoxel incoherent motion (IVIM) diffusion-weighted (DW) and blood oxygen level dependent (BOLD) magnetic resonance imaging (MRI) to assess rat renal function using a 1.5T clinical platform.

**Methods:**

Multiple b-value DW and BOLD MR images were acquired from adult rats using a parallel clinical coil arrangement, enabling quantitation of the apparent diffusion coefficient (ADC), IVIM-derived diffusion coefficient (D), pseudodiffusion coefficient (D*) and perfusion fraction (f), and the transverse relaxation time T_2_*, for whole kidney, renal cortex, and medulla. Following the acquisition of two baseline datasets to assess measurement repeatability, images were acquired following i.v. administration of hydralazine, furosemide, or angiotensin II for up to 40 min.

**Results:**

Excellent repeatability (CoV <10 %) was observed for ADC, D, f and T_2_* measured over the whole kidney. Hydralazine induced a marked and significant (*p* < 0.05) reduction in whole kidney ADC, D, and T_2_*, and a significant (*p* < 0.05) increase in D* and f. Furosemide significantly (*p* < 0.05) increased whole kidney ADC, D, and T_2_*. A more variable response to angiotensin II was determined, with a significant (*p* < 0.05) increase in medulla D* and significant (*p* < 0.05) reduction in whole kidney T_2_* established.

**Conclusions:**

Multiparametric MRI, incorporating quantitation of IVIM DWI and BOLD biomarkers and performed on a clinical platform, can be used to monitor the acute effects of vascular and tubular modulating drugs on rat kidney function in vivo. Clinical adoption of such functional imaging biomarkers can potentially inform on treatment effects in patients with renal dysfunction.

**Electronic supplementary material:**

The online version of this article (doi:10.1186/s12882-016-0356-x) contains supplementary material, which is available to authorized users.

## Background

Decreased renal perfusion and medullary oxygenation are considered predisposing factors to the onset of acute kidney injury [1]. Furthermore, renal medullary oxygenation has been implicated in hypertension and diabetic nephropathy [2, 3]. Non-invasive imaging techniques such as magnetic resonance imaging (MRI) are being actively evaluated pre-clinically to advance the understanding of renal microcirculation and pathophysiology in vivo, with a strong emphasis on establishing their robustness for clinical translation [4].

MRI is an important tool for clinical disease assessment and treatment response. In addition to exquisite soft tissue contrast and anatomic detail, advanced functional MRI techniques, such as diffusion-weighted imaging (DWI) and blood oxygenation level-dependent (BOLD) MRI now provide a means of defining quantitative biomarkers to inform on biologically relevant structure-function relationships in tissues, enabling an understanding of their behaviour and spatial distribution [5].

In nephrology, application of a monoexponential model to DW MR images of increasing diffusion-weighting yields the apparent diffusion coefficient (ADC), shown to decrease as a consequence of acute tubular necrosis, renal artery stenosis, and acute and chronic renal failure, as well as post-transplant kidney rejection [6–8]. The biexponential intravoxel incoherent motion (IVIM) model reports two distinct diffusion constants as fractional components; the pseudodiffusion constant D*, of fractional volume f, associated with a faster incoherent flow component, and D which reflects random tissue water diffusion [9]. Highly vascular tissues such as the kidney show non-monoexponential signal decay with increasing diffusion weighting (b-value) [10, 11], thought to reflect a rapidly decaying signal component visible at lower b-values. The physiological contribution towards this fast diffusion component is however complex because of the presence of a tubular flow fraction, which may exhibit similar diffusion characteristics to the vascular contribution. Using IVIM DWI may improve the understanding of the contribution of renal tubular flow towards the diffusion signal [12].

BOLD MRI has been extensively used both pre-clinically and clinically to assess renal medullary oxygenation, using deoxyhaemoglobin as an endogenous contrast agent. Deoxyhaemoglobin is paramagnetic and creates magnetic susceptibility perturbations around blood vessels, decreasing the MRI transverse relaxation time T_2_*. As the oxygenation state of haemoglobin is related to the arterial blood p_a_O_2_, which is in equilibrium with tissue pO_2_, changes in renal T_2_* are used to infer alterations in renal pO_2_ [13].

Multiparametric MRI strategies, enabling the investigation of several imaging biomarkers in the same imaging session, are being increasingly exploited to provide additional mechanistic insight. The combination of IVIM DWI with BOLD imaging has the potential to illuminate the relative sensitivity of the biexponential DWI signal to vascular versus tubular flow, by observing and correlating the changes in IVIM parameters with T_2_* [14, 15]. In this study, the repeatability of, and the effects of established pharmacological/physiological interventions known to modulate renal vascular flow and/or renal tubular excretion on, IVIM DWI and BOLD MRI biomarkers were investigated in the rat kidney in vivo. This multiparametric imaging strategy was applied on a clinical 1.5T MRI platform, using standard clinical hardware, and used the known actions of hydralazine, furosemide, and angiotensin II to probe the spatial relationship between blood oxygenation, blood flow, vascular fraction, and tubular flow within the kidney.

## Methods

### Animal preparation

This study was performed in accordance with the local ethical review panel, the UK Home Office Animals (Scientific Procedures) Act 1986, the United Kingdom National Cancer Research Institute guidelines for the welfare of animals in cancer research and the ARRIVE (animal research: reporting in vivo experiments) guidelines [16, 17] (see Additional file 1 for ARRIVE checklist). Female Sprague-Dawley rats (*n* = 4 total, three randomised to each imaging session, 250–300 g, Charles River, Margate, UK) were anaesthetised with a 4 ml/kg intraperitoneal injection of fentanyl citrate (0.315 mg/ml) plus fluanisone (10 mg/ml (Hypnorm; Janssen Pharmaceutical Ltd. High Wycombe, UK)), midazolam (5 mg/ml (Hypnovel; Roche)), and water (1:1:2). A lateral tail vein was cannulated with a heparinised 27G butterfly catheter (Venisystems, Hospira, Royal Leamington Spa, UK) to enable the remote administration of drugs.

### MRI acquisition

MRI was performed on a MAGNETOM Avanto 1.5T, 60 cm horizontal-bore clinical scanner (Siemens Healthcare, Erlangen, Germany). The rat was secured supine along the magnet axis centred on top of a small-loop temporomandibular joint (TMJ) coil, and placed within the multi-element head receiver coil. Animals were supported using an insulating vacuum beanbag; use of the beanbag was sufficient to retain the animal’s body heat through the imaging session, as well as to prevent excessive movement. Elements of the head coil array were used in parallel with the small-loop coil during all acquisitions. Scans were performed in the coronal plane, with multiple imaging sections covering both kidneys. Morphological images were obtained for anatomical localisation, with multi-slice fast spin-echo sequence, repetition time (TR) = 800 ms, echo time (TE) = 9.6 ms, voxel size 0.5 mm^2^ in-plane, 1 mm thick slice, matrix 256 × 149, scan time 4 min 30 s.

For DWI studies, images were acquired in free-breathing using a 2D EPI sequence with spectral attenuated inversion recovery (SPAIR) fat suppression, TR = 2.1 s and TE = 71 ms, with 9 b-values (0, 20, 40, 60, 80, 100, 200, 400, 800 s/mm^2^ trace-weighted), isotropic voxel size 1.5 mm^3^, matrix 72 × 72, parallel imaging factor of 2 and 18 signal averages (acquisition time 16 min). The imaging sequence mirrors the b-values used for clinical diffusion-weighted imaging in patients. DW images were thus acquired prior to and 40 min after administration of drug, or with 40 min spacing for repeatability data; based on extant literature it was predicted that this time window would be sufficient to capture the long action of administered agents [18].

BOLD imaging was performed using a multiple gradient echo sequence, with TR = 300 ms, TE = 5, 10, 20, 30, and 40 ms, fat suppression, parallel imaging factor 2, voxel size 0.6 × 0.6 mm in-plane and 5 mm slice thickness. A 128 × 128 matrix was acquired and interpolated to 256 × 256. The flip angle was 25°, with 12 long-term signal averages, giving an acquisition time of 4 min 30 s. BOLD data were acquired at baseline, and repeated three times (scan time for all BOLD measurements 18 min) immediately following administration of drug.

For each rat, imaging was performed twice in the same session without any drug administration to determine measurement repeatability and coefficients of variation (CoV), and before and after administration of each drug. The animals were allowed to fully recover before any subsequent imaging session. Rats were administered with either hydralazine (5 mg/kg, Sigma-Aldrich, Poole, UK), furosemide (5 mg/kg, Sigma-Aldrich), both given as a bolus injection, or angiotensin II (Sigma-Aldrich), with a half-life of 16 ± 1 s [19], infused at 0.5 μg/min/kg using a power injector. The known acute effects of these drugs are vasodilation, diuresis, and vasoconstriction, respectively, and thus expected to induce MRI signal changes in renal perfusion/tubular flow over the imaging timecourse.

### MRI data analysis

**Fig. 1:**
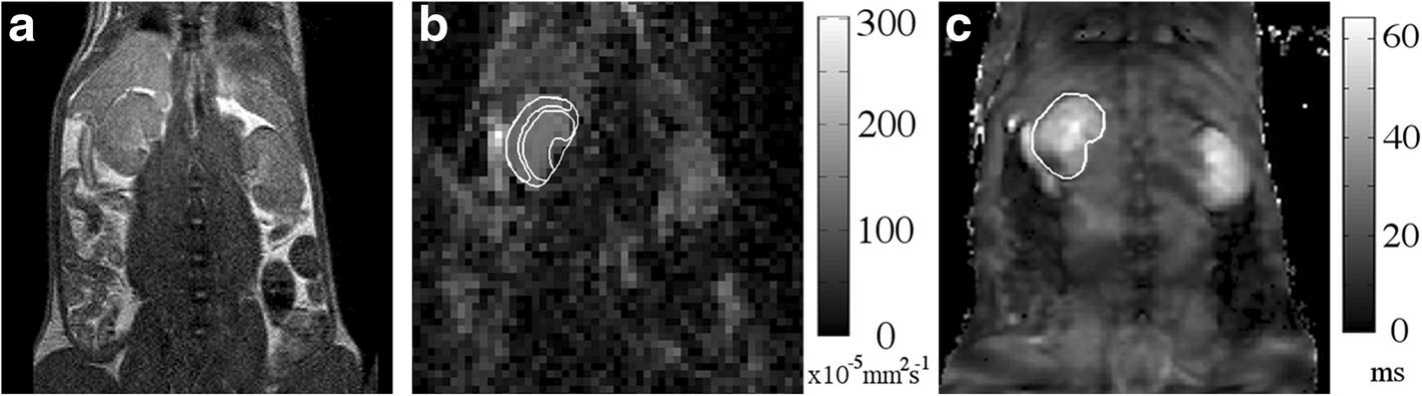
Representative MRI images. Typical images from **a**) T_2_-weighted anatomical scan, with corresponding **b**) ADC map and **c**) T_2_* map from a matched slice, including example ROIs to indicate the selections for analysis of renal cortex and medulla

DW images were reviewed and analysed by an MR scientist with 4 years experience in conducting pre-clinical MR studies. DW-MRI and BOLD images were processed using proprietary software (ADEPT, The Institute of Cancer Research, UK). DWI ROIs were drawn on the central slice of each kidney through the equator plane on the calculated S_0_ image, using anatomic T_2_-weighted and b = 800 s/mm^2^ images for reference, around the entire renal outline (91 ± 12 voxels) of both kidneys, and within regions of the cortex and medulla (28 ± 7 voxels), defined as the single voxel outline of the kidney and the region inside, respectively (see Fig. 1 for example ROIs). For each analysis, fitting for perfusion-insensitive ADC (×10^−5^ mm^2^s^−1^) was performed using images for b = 200 s/mm^2^ and above, with a single-exponential model and a Levenberg-Marquardt algorithm. In tissues where the monoexponential ADC model is known to be a poor model, excluding the b-values known to be most sensitive to perfusion minimizes the dependence of resultant ADC on the exact choice of b-values used [20]. All b-value images were used for IVIM curve fitting; initial parameter estimates were obtained by fitting a monoexponential model at b > 200 s/mm^2^ to give an estimate of D, and projecting back to b = 0 to provide an initial estimate of the perfusion fraction f. Following this, an adaptation of Bayesian approaches [21, 22] was applied to all b-value data to derive final estimates of D (×10^−5^ mm^2^ s^−1^), f (%), and the pseudodiffusion coefficient D* (×10^−2^ mm^2^ s^−1^). The product estimate fD* (×10^−4^ mm^2^s^−1^), associated with flow, was also calculated; the adapted Bayesian approach assumed Gaussian data errors and uniform prior distributions for all unknown parameters.

For BOLD images, partial volume effects from slice thickness precluded accurate separation of the renal cortex and medulla, hence ROIs were drawn around the central slice of each kidney in the equatorial plane (516 ± 58 voxels). Single-exponential fitting with the Levenberg-Marquardt algorithm yielded estimates for T_2_*; sufficient signal to noise (SNR >20) was recorded at all echo times, and noise was not included in the model.

For all analyses, the median values for each ROI were reported, reducing effects from voxels where fitting did not converge. In cases where the slice positioning or signal precluded confident drawing of the ROI, the kidney was excluded from analysis (*n* = 1, furosemide).

For statistical comparison, significance of results was assessed using non-parametric tests at 5 % using a Wilcoxon paired rank sum for DWI measurements, with a one-way ANOVA for the repeated BOLD measurements. The Pearson correlation statistic was calculated for values of the related diffusion parameters ADC and D.

## Results

The use of parallel clinical coils on the 1.5T platform provided sufficient signal for functional MRI of the rat kidney. Representative anatomical T_2_-weighted images, and associated parametric ADC and T_2_* maps calculated from the same rat, with the renal cortex and medulla delineated in ADC map and whole kidney on T_2_*, are shown in Fig. 1.

### DW MRI model comparison

**Fig. 2:**
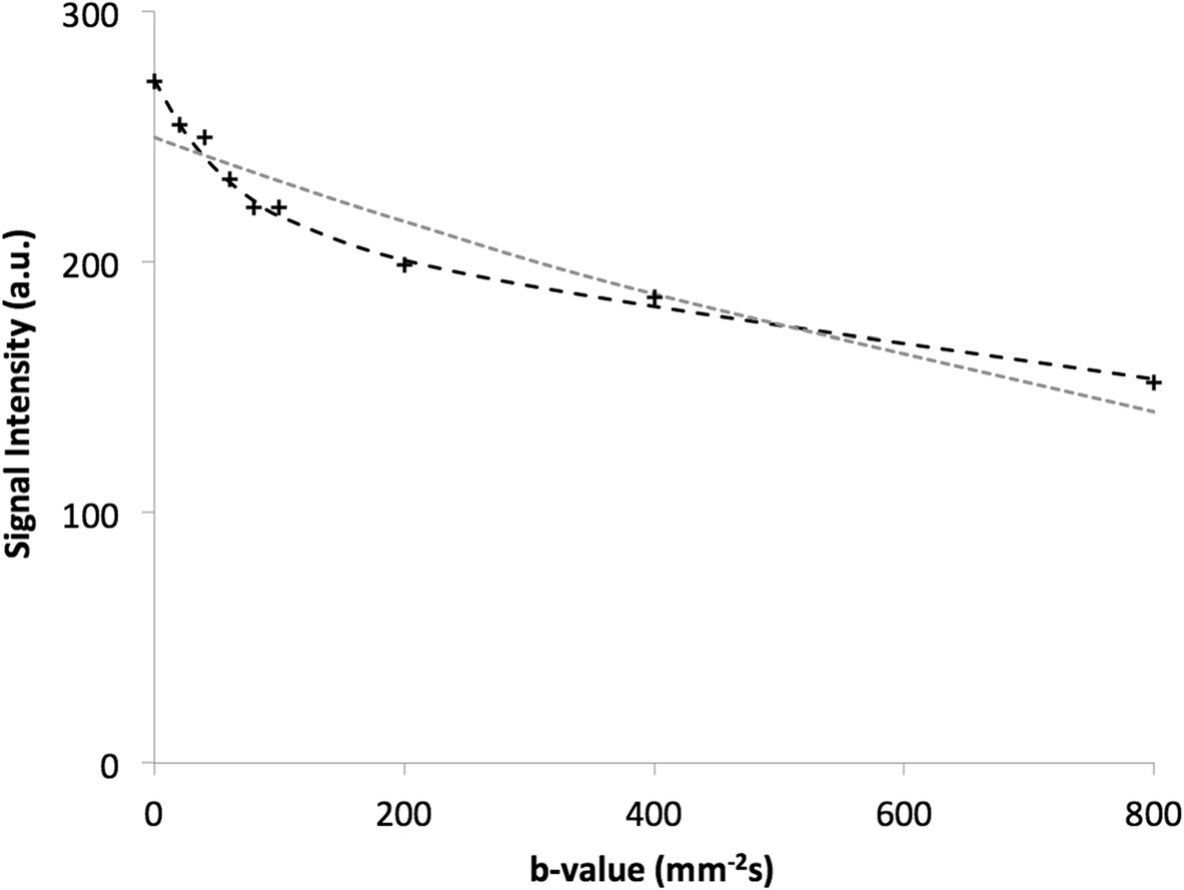
Representative diffusion signal plots. Signal intensity (arbitrary units) plotted against diffusion b-value for a single voxel acquired from one rat kidney, showing the fitted bi-exponential IVIM model (*black line*), favoured in the presence of a perfusion contribution, and single exponential ADC model (*grey line*)

In all cases, non-monoexponential behaviour was evident in the kidneys from plots of signal intensity against b-value. The baseline signal intensity curve against b-value for a representative renal voxel is shown in Fig. 2. Non-monoexponential decay was evident, with the overlaid fitted curves for monoexponential ADC and biexponential IVIM models for all b-values clearly favouring the inclusion of the pseudodiffusion/perfusion component. The median residuals from fitting the ADC and IVIM models for the whole kidney in the entire study population, taken as a proxy for quality of model fitting and normalised to degrees of freedom, were 24.3 and 12.3 a.u., respectively.

Taking the diffusion parameter D from the IVIM model, and comparing to the perfusion-insensitive ADC (calculated using b = 200 mm^−2^s and higher), an expected good agreement was found both at baseline and following drug challenge (Tables 1 and 2). Pearson correlation coefficients for whole kidney ROIs from all studies were 0.86 at baseline and 0.98 post-challenge.

**Table 1:** Repeatability measurements for ADC and IVIM diffusion models

	Kidney			Cortex			Medulla		
	Scan 1	Scan 2	CoV (%)	Scan 1	Scan 2	CoV (%)	Scan 1	Scan 2	CoV (%)
f (%)	26.9 ± 1.1	27.7 ± 2.3	18.7	27.1 ± 3.1	29.7 ± 3.7	24.5	23.4 ± 1.9	25.3 ± 3.5	22.5
D (10^−5^ mm^2^s^−1^)	124.5 ± 7.5	113.4 ± 4.4	6.2	105.0 ± 5.2	96.7 ± 6.9	12.4	133.0 ± 10.6	119.4 ± 7.3	7.4
ADC (10^−5^ mm^2^s^−1^)	101.9 ± 7	105.8 ± 4.4	9.3	85.5 ± 6.7	89.7 ± 7.3	17.5	111.3 ± 7.3	117.6 ± 5.5	11.9
D* (10^−2^ mm^2^s^−1^)	4.0 ± 0.4	5.4 ± 0.8	24.3	3.7 ± 0.5	4.5 ± 0.8	32.8	4.2 ± 0.5	5.6 ± 0.9	27.4
fD* (10^−4^ mm^2^s^−1^)	95.2 ± 7.3	151.2 ± 33.3	30.6	81.8 ± 6.8	129.5 ± 29.2	41.6	86.0 ± 9.0	150.4 ± 42.3	39.9

**Table 2:** Fitted ADC and IVIM parameter estimates following vasomodulator challenge

		Kidney		Cortex		Medulla	
	Challenge	pre	post	pre	post	pre	post
	Hydralazine	10.9 ± 0.7	15.1 ± 0.8^a^	17.4 ± 2.1	24.4 ± 2.6^a^	7.2 ± 0.8	8.3 ± 1.3
f	Furosemide	*12.0 ± 1.4*	*10.1 ± 1.0* ^*b*^	17.1 ± 1.0	12.0 ± 1.5^a^	*9.6 ± 1.7*	*8.2 ± 1.3* ^*b*^
(%)	Angiotensin II	19.3 ± 1.5	18.7 ± 1.6	23.9 ± 2.9	21.6 ± 2.1	15.3 ± 1.5	13.3 ± 1.3
	Hydralazine	133.9 ± 5.9	100.2 ± 6.6^a^	130.3 ± 7.2	96.8 ± 8.8^a^	139.3 ± 4.0	109.6 ± 5.4^a^
D	Furosemide	118.8 ± 5.8	138.8 ± 5.0^a^	117.5 ± 7.7	137.4 ± 7.9^a^	123.5 ± 5.4	141.6 ± 4.3^a^
(10^−5^ mm^2^s^−1^)	Angiotensin II	118.8 ± 7.0	115.0 ± 4.4	116.4 ± 7.3	110.7 ± 4.7	129.1 ± 8.7	123.8 ± 5.7
	Hydralazine	130 ± 6.0	97.9 ± 7.1^a^	126.6 ± 6.5	87.9 ± 8.9^a^	139.0 ± 4.0	111.8 ± 6.2^a^
ADC	Furosemide	117.1 ± 5.8^a^	136.2 ± 5.7^a^	114.1 ± 9.1^a^	134.5 ± 8.6^a^	122.7 ± 5.5^a^	141.1 ± 5.6^a^
(10^−5^ mm^2^s^−1^)	Angiotensin II	110.9 ± 7.7	110.9 ± 3.9	103.1 ± 8.1	102.2 ± 4.7	126.0 ± 8.8	122.2 ± 2.7
	Termination	91.3 ± 9.5	68.0 ± 4.3^a^	84.1 ± 9.8	60.5 ± 5.3^a^	100.7 ± 10.2	79.0 ± 10.7
	Hydralazine	4.0 ± 0.2	5.0 ± 0.2^a^	3.3 ± 0.4	5.0 ± 0.2^a^	4.3 ± 0.3	4.9 ± 0.2^a^
D*	Furosemide	3.6 ± 0.2	3.8 ± 0.1	3.2 ± 0.3	3.7 ± 0.2	3.8 ± 0.2	3.6 ± 0.2
(10^−2^ mm^2^s^−1^)	Angiotensin II	4.3 ± 0.5	5.1 ± 0.3	4.2 ± 0.4	4.5 ± 0.3	*4.3 ± 0.6*	*5.2 ± 0.4* ^*b*^
	Hydralazine	38.7 ± 1.7	65.6 ± 1.9^a^	48.1 ± 3.4	96.7 ± 9.2^a^	25.2 ± 0.9	35.6 ± 3.7^a^
fD*	Furosemide	38.4 ± 5.0	32.5 ± 3.2	48.4 ± 5.0	37.8 ± 4.3^a^	29.9 ± 4.7	25.0 ± 2.7
(10^−4^ mm^2^s^−1^)	Angiotensin II	71.2 ± 11.5	80.8 ± 10.8	76.8 ± 9.4	83.0 ± 12.9	59.0 ± 12.3	62.8 ± 9.5

Data are reported as mean of median ROI values ± standard error

^a^indicates *p* < 0.05 against baseline

^b^indicates *p* < 0.1 against baseline

### Repeatability

**Fig. 3:**
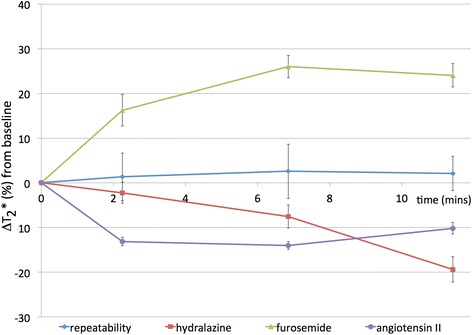
Timecourse of intrinsic susceptibility contrast measurements. Graphs showing percentage change in renal T_2_* following administration of saline or vasomodulators. Data points are mean ± 1 standard error of median ROI values

Good measurement repeatability of both diffusion and BOLD parameters was determined. The percentage change in fitted T_2_* in the ROI from baseline revealed no significant deviation, less than 2.6 %, over the experimental timecourse (CoVs from each time point to the next were less than 1 %), and was small relative to the effects determined following drug challenges (Fig. 3, Table 3). Diffusion characteristics also showed no substantive change, except for D, which showed a significant decrease (*p* < 0.05) for the whole kidney ROI, although this result was not mirrored in the ADC fitting, which showed no significant change (Table 1). CoVs were comparable for ADC and D in each of the ROIs considered, indicating the robustness of fitting true diffusion, with the fast pseudo-diffusion constant D* from the IVIM model having a substantially higher CoV.

**Table 3:** Renal T_2_* (ms) for successive timepoints following challenge

Time	0	2m15s	6m51s	11m24s
Repeatability	32.8 ± 1.4	33.3 ± 1.5	33.7 ± 1.5^a^	33.5 ± 1.3
Hydralazine	37.7 ± 2.0	36.7 ± 2.3	34.5 ± 1.5	30.2 ± 1.4^a^
Furosemide	38.5 ± 2.2	44.7 ± 2.4^ab^	48.5 ± 2.8^ab^	47.8 ± 3.0^a^
Angiotensin II	34.5 ± 2.4	29.8 ± 1.9^ab^	29.4 ± 1.1^a^	30.7 ± 1.1^a^

Data are reported as mean of median ROI values ± 1 standard error of the mean

^a^indicates *p* < 0.05 against baseline

^b^indicates *p* < 0.05 against previous timepoint

### Hydralazine

**Fig. 4:**
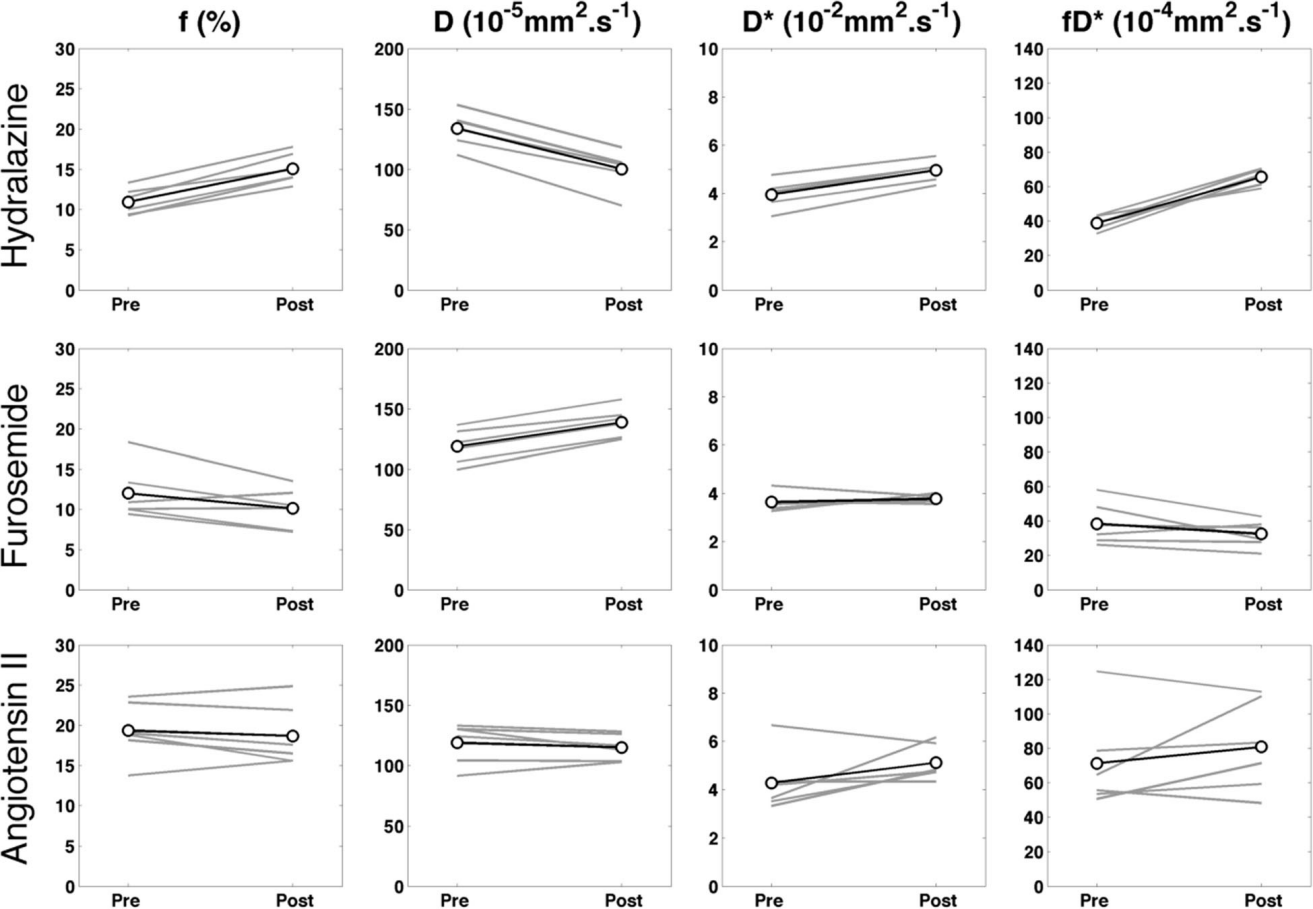
Sensitivity of diffusion-weighted imaging parameters. Individual ladder plots (*grey*) and cohort means (*black*) for diffusion parameters derived from whole kidney ROIs, determined prior to and following administration of hydralazine, furosemide or angiotensin II

**Fig. 5:**
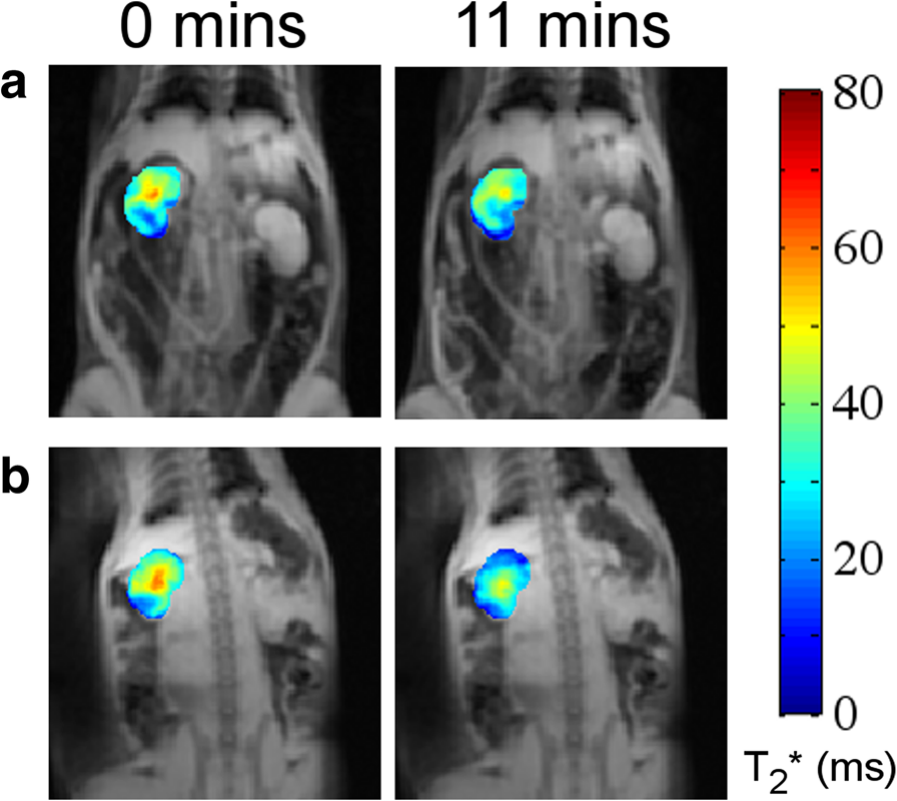
Repeatability measurements show parameter stability. Functional T_2_* maps (overlaid on TE = 5 ms image) for whole kidney (**a**) with no intervention showing measurement repeatability, and **b**) prior to and 11 min following intravenous administration of hydralazine

Hydralazine induced significant changes in all the diffusion parameters (*p* < 0.05) for all regions, except the medulla vascular fraction (f) among the fitted IVIM diffusion parameters (Table 2). Ladder plots of median results for individual whole-kidney ROIs showed remarkable consistency, with f, D*, and fD* increasing, and D decreasing (Fig. 4; perfusion-insensitive ADC closely matched D for response). For the cortex and medulla ROIs the same trend was observed, with the cortex having higher f and lower ADC than the medulla. The highly vascular renal cortex appeared to account for the majority of the increase in f observed at the whole-kidney level. BOLD MRI showed a progressive decrease in T_2_* after administration of hydralazine, becoming significant (*p* < 0.05) after 11 min (Table 3). Representative T_2_* maps from a repeatability scan (no intervention), together with maps acquired prior to and post-hydralazine, are shown in Fig. 5, clearly showing the pharmacologically-induced change in transverse relaxation.

### Furosemide

Diffusion parameter response to furosemide was more wide-ranging than the response to hydralazine, with greater variations between subjects (Fig. 4), but with no significant change in vascular fraction overall. In contrast, the D and perfusion-insensitive ADC increased significantly and consistently in all ROIs, but D* was unaffected (Fig. 4). The temporal BOLD response post administration was pronounced, with an immediate and significant (*p* < 0.05) increase in T_2_* that reached over 125 % of baseline value at 6 min and remained elevated for the final time point.

### Angiotensin II

Infusion of angiotensin II induced marked inter-renal variation in DWI response, with a significant (*p* < 0.05) increase determined in medulla D* only (Fig. 4). This was associated with a significant (*p* < 0.05 for baseline against post-administration time points) yet potentially transient decrease in T_2_*.

## Discussion

In this study, we developed and applied a multi-parametric MRI strategy to evaluate the combination of DW and BOLD MRI for the assessment of rat renal function in vivo on a 1.5T clinical platform. IVIM DWI allows for the estimation of a fast component of water diffusivity (f, D*, and fD*) related to renal perfusion and tubular excretion, in addition to measuring tissue diffusivity (D). A challenge of using IVIM DWI to measure renal function is the difficulty in decoupling vascular and tubular contributions, as the two are intimately linked through autoregulation to maintain homeostasis. Additional mechanistic insight may be possible by combining IVIM DWI data with quantitative T_2_* measurements using BOLD MRI, which reflects renal blood volume and tissue oxygenation. Studies have shown that increased T_2_* in the kidneys can result from increased hypoxia from higher tubular metabolism or increased blood volume [23, 24]. Combining IVIM DWI with T_2_* measurements allows corroboration of mutual information to explain changes in renal physiology with pharmacological/physiological intervention.

Repeatability measurements are critical for providing confidence in observed changes following experimental intervention, but are not routinely performed in pre-clinical imaging investigations. In this study, we first established the repeatability of the quantitative MRI biomarkers, with low CoVs for repeated measures demonstrating that the functional parameters did not suffer from instability over the imaging timecourse. Comparison of the perfusion-insensitive ADC and the IVIM parameter D showed good agreement both prior to and during challenge, suggesting that perfusion effects are mostly removed at b-values >200 mm^−2^s. Although the validity of this assumption will vary with different tissues, this demonstrates the usefulness of the Bayesian fitting method used herein for removing this assumption and fitting the whole dataset, rather than the more common two-stage fitting of IVIM data. The use of clinical vendor surface coils used in parallel with volume coils provided sufficient signal and resolution to measure the MRI biomarkers with good repeatability. Unsurprisingly, measurement repeatability was poorer for the perfusion sensitive parameters compared with the perfusion-insensitive parameters and T_2_*.

We then proceeded to evaluate the effects of drugs known to modulate renal vascular and tubular function. Hydralazine is a well-characterised systemic vasodilator that relaxes vascular smooth muscle; the increased vascular fraction determined in the IVIM model, and reduction in T_2_*, is consistent with a hydralazine-induced increase in (deoxygenated) renal blood volume. Furthermore, the associated reduction in blood flow induces a compensatory increase in cardiac output to maintain blood pressure [25], resulting in the observed increase in D* with net increased blood flow. To maintain fluid homeostasis, the kidney may also increase tubular transport, the resulting increase in oxygen consumption also contributing to the reduction in T_2_*. The decrease in the perfusion-insensitive ADC and D can be interpreted as a consequence of dehydration of the interstitial space.

Furosemide is a loop diuretic, inhibiting water reabsorption in the nephron by blocking the sodium-potassium-chloride co-transporter in the ascending limb of the loop of Henlé. The rapid and significant increase in T_2_* seen herein is consistent with previous reports and the known effects of furosemide on reducing renal blood volume and decreasing oxygen consumption [14, 26, 27]. A reduction in perfusion fraction in the renal cortex was also observed, suggesting a reactive decrease in vascular flow, and also consistent with a reduction in blood volume. Interestingly, the perfusion-insensitive ADC and D significantly increased, consistent with an increase in renal water content within the tubules due to diuretic effects. This contrasts with previous studies showing either no change, or a reduction in renal water diffusivity, in response to furosemide [14, 28].

Angiotensin II is a naturally-occurring hormone with a complex role within the renal renin-angiotensin system (RAS) [29]. The hormone has a direct effect on the proximal tubules to increase Na^+^ reabsorption, and has a convoluted and variable effect on glomerular filtration and renal blood flow. Increases in systemic blood pressure will maintain renal perfusion pressure; however, constriction of the afferent and efferent glomerular arterioles can reduce renal blood flow. The effect on the efferent arteriolar resistance tends to increase glomerular capillary hydrostatic pressure and maintain glomerular filtration rate. In the present study, the initial reduction in T_2_* is consistent with an acute reduction in blood volume and tissue oxygenation, the suggestion of a recovery towards baseline may reflect a reactive response to drug-induced vasoconstriction. A similar transient T_2_* response to angiotensin II has been reported in human kidney [30]. The DWI data revealed no significant changes, which supports the concept of an acute effective homeostatic response, and the absence of any vascular response in the IVIM parameters.

Animal welfare and ways of reducing animal usage is an important consideration for all research bioscientists; non-invasive and longitudinal imaging methods that incorporate repeatability measurements can reduce the number of animals by using each as its own control, harnessing statistical power through the use of paired statistical tests in small animal cohorts, and information that may relate better to that observed in clinical assessment of therapy efficacy. Clinical MRI scanners are being increasingly used for pre-clinical imaging studies. Compared to relatively expensive dedicated small-bore animal MRI systems, major vendors sell and distribute far more clinical scanners; consequences of this include the continuing development and availability of superior hardware and standardised pulse sequences on these platforms. Pre-clinical studies performed on clinical platforms also provide evidence supporting the clinical relevance of advanced diffusion modelling and data acquisition. Most clinical scanners operate at between 1.5 and 3 Tesla, and thus have lower signal-to-noise (SNR) levels than pre-clinical systems, with reduced image quality if conventional clinical imaging coils are used. This is exacerbated particularly when imaging the small fields-of-view necessary when using rodents, but with a high enough resolution to be able to acquire meaningful functional data. One approach for increasing SNR is to use small, dedicated receiver coils, such as the TMJ coil used herein, designed to fit closely to the object of interest, giving a better coupling between the object and coil with a corresponding increase in signal and thus improved image quality [31]. Here we have shown the sensitivity and stability of parallel imaging using standard vendor coils on a clinical 1.5T MR system for conducting rodent renal studies, and demonstrate the sensitivity of resulting DWI and BOLD MRI biomarkers to the effects of several vasomodulators. This experimental arrangement extends the available scope for performing pre-clinical studies with existing clinical hardware, and confers the advantages associated with increased access to clinical scanners for pre-clinical studies.

Some limitations in this study are clear. Firstly, due to the voxel size achievable on clinical 1.5T MR systems, we were limited in our ability to reliably draw smaller ROIs to interrogate regions within the kidney that may contain differential vascular/tubular components, and this was particularly true for DWI where signal is actively attenuated by the sensitising gradients. Hence, analysis was necessarily made over larger regions to ensure adequate image SNR. Secondly, the dynamic imaging protocol precluded the use of other complex invasive measurements to provide additional validation of our observations against other physiological measures over the experimental period.

## Conclusions

We have shown that IVIM DWI and T_2_* measurements are feasible on a 1.5T clinical MR system to monitor the acute effects of vascular and tubular modulating drugs on rat kidney function in vivo. Water diffusion in kidneys exhibits bi-exponential behaviour, with the fast diffusion component at low b-values reflecting both vascular and tubular flow. Multi-parametric MRI strategies combining IVIM DWI with T_2_* measurements allows mutual corroboration of pharmacological interventions. The administration of intravenous hydralazine, furosemide, or angiotensin showed differential effects on IVIM DWI and BOLD MRI biomarkers in vivo, and highlights the potential of these techniques to study the effects of drugs that modulate renal function in humans, so as to better understand their treatment effects in patients with renal dysfunction.

## Additional file

Additional file 1: ARRIVE Checklist. (DOCX 42 kb)

## Abbreviations

ADC: Apparent diffusion coefficient
ANOVA: Analysis of variance
ARRIVE: Animal research: reporting in vivo experiments
BOLD: Blood oxygenation level dependent
CoV: Coefficient of variation
DWI: Diffusion-weighted imaging
DW-MRI: Diffusion-weighted MRI
EPI: Echo-planar imaging
IVIM: Intravoxel incoherent motion
MR: Magnetic resonance
MRI: Magnetic resonance imaging
RAS: Renin-angiotensin system
ROI: Region of interest
SNR: Signal-to-noise ratio
SPAIR: Spectral attenuated inversion recovery
TE: Echo time
TMJ: Temporomandibular joint
TR: Repetition time
