# Supplementary material for: Modulation of renal oxygenation and perfusion in rat kidney monitored by quantitative diffusion and blood oxygen level dependent magnetic resonance imaging on a clinical 1.5T platform
Source: BMC Nephrol. 2016 Oct 3;17:142. doi: 10.1186/s12882-016-0356-x (PMC5048450; doi:10.1186/s12882-016-0356-x)
Supplement: Additional file 1: — ARRIVE Checklist. (DOCX 42 kb) [file 12882_2016_356_MOESM1_ESM.docx]

**ARRIVE Checklist**

**TITLE**

**1 Provide as accurate and concise a description of the content of the article as possible.**

Modulation of Renal Oxygenation and Perfusion in Rat Kidney Monitored by Quantitative Diffusion and Blood Oxygen Level Dependent Magnetic Resonance Imaging on a Clinical 1.5T Platform

**ABSTRACT**

**2 Provide an accurate summary of the background, research objectives (including details of the species or strain of animal used), key methods, principal findings, and conclusions of the study.**

*Background:* To investigate the combined use of intravoxel incoherent motion (IVIM) diffusion-weighted (DW) and blood oxygen level dependent (BOLD) magnetic resonance imaging (MRI) to assess rat renal function using a 1.5T clinical platform.

*Methods:* Multiple b-value DW and BOLD MR images were acquired from adult rats using a parallel clinical coil arrangement, enabling quantitation of the apparent diffusion coefficient (ADC), IVIM-derived diffusion coefficient (D), pseudodiffusion coefficient (D*) and perfusion fraction (f), and the transverse relaxation time T_2_*, for whole kidney, renal cortex, and medulla. Following the acquisition of two baseline datasets to assess measurement repeatability, images were acquired following i.v. administration of hydralazine, furosemide, or angiotensin II for up to 40 minutes.

*Results:* Excellent repeatability (CoV<10%) was observed for ADC, D, f and T_2_* measured over the whole kidney. Hydralazine induced a marked and significant (p<0.05) reduction in whole kidney ADC, D, and T_2_*, and a significant (p<0.05) increase in D* and f. Furosemide significantly (p<0.05) increased whole kidney ADC, D, and T_2_*. A more variable response to angiotensin II was determined, with a significant (p<0.05) increase in medulla D* and significant (p<0.05) reduction in whole kidney T_2_* established.

*Conclusions:* Multiparametric MRI, incorporating quantitation of IVIM DWI and BOLD biomarkers and performed on a clinical platform, can be used to monitor the acute effects of vascular and tubular modulating drugs on rat kidney function *in vivo*. Clinical adoption of such functional imaging biomarkers can potentially inform on treatment effects in patients with renal dysfunction.

**INTRODUCTION**

**Background**

**3 a. Include sufficient scientific background (including relevant references to previous work) to understand the motivation and context for the study, and explain the experimental approach and rationale.**

**b. Explain how and why the animal species and model being used can address the scientific objectives and, where appropriate, the study’s relevance to human biology.**

Decreased renal perfusion and medullary oxygenation are considered predisposing factors to the onset of acute kidney injury, with renal medullary oxygenation implicated in hypertension and diabetic nephropathy. Non-invasive imaging techniques such as magnetic resonance imaging (MRI) are being actively evaluated pre-clinically to advance the understanding of renal microcirculation and pathophysiology *in vivo*, with a strong emphasis on establishing their robustness for clinical translation. MRI is an important tool for clinical disease assessment and treatment response, providing quantitative biomarkers to inform on biologically relevant structure-function relationships in tissues.

Multiparametric MRI strategies, enabling the investigation of several imaging biomarkers in the same imaging session, are being increasingly exploited to provide additional mechanistic insight. The combination of IVIM DWI with BOLD imaging has the potential to illuminate the relative sensitivity of the biexponential DWI signal to vascular versus tubular flow, by observing and correlating the changes in IVIM parameters with T_2_*.

**Objectives**

**4 Clearly describe the primary and any secondary objectives of the study, or specific hypotheses being tested.**

The aim of this study is to assess the repeatability of, and the effects of established pharmacological/physiological interventions known to modulate renal vascular flow and/or renal tubular excretion on, IVIM DWI and BOLD MRI biomarkers in the rat kidney *in vivo*.

**METHODS**

**Ethical statement**

**5 Indicate the nature of the ethical review permissions, relevant licenses (e.g. Animal [Scientific Procedures] Act 1986), and national or institutional guidelines for the care and use of animals, that cover the research.**

This study was performed in accordance with the local ethical review panel, the UK Home Office Animals (Scientific Procedures) Act 1986, the United Kingdom National Cancer Research Institute guidelines for the welfare of animals in cancer research and the ARRIVE (animal research: reporting in vivo experiments) guidelines.

**Study design**

**6 For each experiment, give brief details of the study design, including:**

**a. The number of experimental and control groups.**

**b. Any steps taken to minimise the effects of subjective bias when allocating animals to treatment (e.g., randomisation procedure) and when assessing results (e.g., if done, describe who was blinded and when).**

**c. The experimental unit (e.g. a single animal, group, or cage of animals).**

**A time-line diagram or flow chart can be useful to illustrate how complex study designs were carried out.**

Total of 4 rats were used, with 3 assigned to each experiment (full recovery allowed between successive experiments). The study included a repeatability assessment, thus allowing the animals to act as their own control and minimise the cohort size.

**Experimental procedures**

**7 For each experiment and each experimental group, including controls, provide precise details of all procedures carried out. For example:**

**a. How (e.g., drug formulation and dose, site and route of administration, anaesthesia and analgesia used [including monitoring], surgical procedure, method of euthanasia). Provide details of any specialist equipment used, including supplier(s).**

**b. When (e.g., time of day).**

**c. Where (e.g., home cage, laboratory, water maze).**

**d. Why (e.g., rationale for choice of specific anaesthetic, route of administration, drug dose used).**

Animals were anaesthetised with a 4 ml/kg intraperitoneal injection of fentanyl citrate (0.315 mg/ml) plus fluanisone (10 mg/ml (Hypnorm; Janssen Pharmaceutical Ltd. High Wycombe, UK)), midazolam (5 mg/ml (Hypnovel; Roche)), and water (1:1:2). A lateral tail vein was cannulated with a heparinised 27G butterfly catheter (Venisystems, Hospira, Royal Leamington Spa, UK) to enable the remote administration of drugs.

For each rat, imaging was performed twice in the same session without any drug administration to determine measurement repeatability and coefficients of variation (CoV), and before and after administration of each drug. The animals were allowed to fully recover before any subsequent imaging session. Rats were administered with either hydralazine (5 mg/kg, Sigma-Aldrich, Poole, UK), furosemide (5 mg/kg, Sigma-Aldrich), both given as a bolus injection, or angiotensin II (Sigma-Aldrich), with a half-life of 16 ± 1 s,[19] infused at 0.5 μg/min/kg using a power injector.

MRI was performed on a MAGNETOM Avanto 1.5T, 60 cm horizontal-bore clinical scanner. The rat was secured supine along the magnet axis, using an insulating vacuum beanbag to both retain body heat and to prevent excessive movement, centred on top of a small-loop temporomandibular joint (TMJ) coil, and placed within the multi-element head receiver coil (coils used in parallel). Scans included morphological images (4 min 30 seconds), diffusion-weighted imaging (16 minutes, performed twice), and BOLD imaging (4 min 30 seconds, performed 4 times).

**Experimental animals**

**8 a. Provide details of the animals used, including species, strain, sex, developmental stage (e.g., mean or median age plus age range), and weight (e.g., mean or median weight plus weight range).**

**b. Provide further relevant information such as the source of animals, international strain nomenclature, genetic modification status (e.g. knock-out or transgenic), genotype, health/immune status, drug- or test naıve, previous procedures, etc.**

Female Sprague-Dawley rats (n=4, 250-300g, Charles River, Margate, UK) were used in this study.

**Housing and husbandry**

**9 Provide details of:**

**a. Housing (e.g., type of facility, e.g., specific pathogen free (SPF); type of cage or housing; bedding material; number of cage companions; tank shape and material etc. for fish).**

**b. Husbandry conditions (e.g., breeding programme, light/dark cycle, temperature, quality of water etc. for fish, type of food, access to food and water, environmental enrichment).**

**c. Welfare-related assessments and interventions that were carried out before, during, or after the experiment.**Animals were housed in individually vented cages (IVC; 2 rats per cage) in a part barrier/IVC facility with Corn Cob bedding, Bed-r ‘Nest nesting, play tunnels and aspen bricks, and free access to 5002 Rodent Diet and RO filtered water (bottle). The facility maintained as 12/12 Light/Dark cycle with a temperature of 21 ± 2 °C.

**Sample size**

**10 a. Specify the total number of animals used in each experiment and the number of animals in each experimental group.**

**b. Explain how the number of animals was decided. Provide details of any sample size calculation used.**

**c. Indicate the number of independent replications of each experiment, if relevant.**

See #6.

**Allocating animals to experimental groups**

**11 a. Give full details of how animals were allocated to experimental groups, including randomisation or matching if done.**

**b. Describe the order in which the animals in the different experimental groups were treated and assessed.**

Animals were randomized to each experiment. Imaging sessions were acquired using hydralazine, furosemide, and angiontensin II in that order, with at least 5 days recovery between. Repeatability was assessed in a separate imaging session to the renal modulators.

**Experimental outcomes**

**12 Clearly define the primary and secondary experimental outcomes assessed (e.g., cell death, molecular markers, behavioural changes).**

Imaging parameters relating to diffusion, pseudodiffusion, and transverse relaxation: ADC, D, f, D*, fD*, T2*.

**Statistical methods**

**13 a. Provide details of the statistical methods used for each analysis.**

**b. Specify the unit of analysis for each dataset (e.g. single animal, group of animals, single neuron).**

**c. Describe any methods used to assess whether the data met the assumptions of the statistical approach.**

ROIs were drawn on the central slice of each kidney through the equator plane on the calculated S_0_ image, using anatomic T_2_-weighted and b=800 s/mm^2^ images for reference, around the entire renal outline of both kidneys, and within regions of the cortex and medulla, defined as the single voxel outline of the kidney and the region inside. Fitting for imaging markers (diffusion, pseudodiffusion, transverse relaxation) was performed on a voxel-by-voxel basis and summary statistics per ROI were reported. The IVIM diffusion model was applied using an adaptation of the Markov Chain Monte Carlo approach as a robust least-squares optimiser, assuming Gaussian data errors and uniform prior distributions for all unknown parameters. For all analyses, the median values for each ROI were reported, reducing effects from voxels where fitting did not converge. In cases where the slice positioning or signal precluded confident drawing of the ROI, the kidney was excluded from analysis (n=1, furosemide). For statistical comparison, significance of results was assessed using non-parametric tests at 5% using a Wilcoxon paired rank sum, and the Pearson correlation statistic was calculated for values of the related diffusion parameters ADC and D.

**RESULTS**

**Baseline data
14 For each experimental group, report relevant characteristics and health status of animals (e.g., weight, microbiological status, and drug- or test-naıve) before treatment or testing (this information can often be tabulated).**

All animals remained healthy throughout.

**Numbers analysed**

**15 a. Report the number of animals in each group included in each analysis. Report absolute numbers (e.g. 10/20, not 50%).**

**b. If any animals or data were not included in the analysis, explain why.**

Three animals were included in each experiment.

**Outcomes and estimation**

**16 Report the results for each analysis carried out, with a measure of precision (e.g., standard error or confidence interval).**

*DW MRI Model Comparison*

In all cases, non-monoexponential behaviour was evident in the kidneys from plots of signal intensity against b-value; median residuals from fitting the ADC and IVIM models for the whole kidney in the entire study population were 24.3 and 12.3 a.u. respectively. An expected good agreement was found both at baseline and following drug challenge for ADC and (IVIM) D. Pearson correlation coefficients for whole kidney ROIs from all studies were 0.87 at baseline and 0.98 post-challenge.

*Repeatability*

Good measurement repeatability of both diffusion and BOLD parameters was determined. The percentage change in fitted T_2_* in the ROI from baseline revealed no greater deviation than 2.6% over the experimental timecourse (CoVs from each time point to the next were less than 1%), and was small relative to the effects determined following drug challenges. Diffusion characteristics also showed no substantive change, except for D, which showed a significant decrease (p < 0.05) for the whole kidney ROI, although this result was not mirrored in the ADC fitting, which showed no significant change. CoVs were smaller for ADC than for D in each of the ROIs considered, indicating the robustness of fitting the simpler diffusion model, with the fast pseudo-diffusion constant D* from the IVIM model having a substantially higher CoV.

*Hydralazine*

Hydralazine induced significant changes in all the diffusion parameters (p < 0.05) for all regions, except the medulla vascular fraction (f) among the fitted IVIM diffusion parameters. Median results for individual whole-kidney ROIs showed remarkable consistency, with f and D* and fD* increasing, and D (also ADC) decreasing. For the cortex and medulla ROIs the same trend was observed, with the cortex having higher f and lower ADC than the medulla. The highly vascular renal cortex appeared to account for the majority of the increase in f observed at the whole-kidney level. BOLD MRI showed a progressive decrease in T_2_* after administration of hydralazine, becoming significant (p < 0.05) after 11 minutes.

*Furosemide*

Diffusion parameter response to furosemide was more wide-ranging than the response to hydralazine, with greater variations between subjects, but no overall change in vascular fraction. In contrast, D and ADC increased significantly and consistently in all ROIs, but D* was unaffected. The temporal BOLD response post administration was pronounced, with an immediate and significant (p < 0.05) increase in T_2_* that reached over 125% of baseline value at 6 minutes and remained elevated for the final time point.

*Angiotensin II*

Infusion of angiotensin II induced marked inter-renal variation in DWI response, with a significant (p < 0.05) increase determined in medulla D* only. This was associated with a significant (p < 0.05) yet potentially transient decrease in T_2_*.

**Adverse events**

**17 a. Give details of all important adverse events in each experimental group.**

**b. Describe any modifications to the experimental protocols made to reduce adverse events.**

There were no adverse events.

**DISCUSSION**

**Interpretation/scientific implications**

**18 a. Interpret the results, taking into account the study objectives and hypotheses, current theory, and other relevant studies in the literature.**

**b. Comment on the study limitations including any potential sources of bias, any limitations of the animal model, and the imprecision associated with the results.**

**c. Describe any implications of your experimental methods or findings for the replacement, refinement, or reduction (the 3Rs) of the use of animals in research.**

In this study, we developed and applied a multi-parametric MRI strategy to evaluate the combination of DW and BOLD MRI for the assessment of rat renal function *in vivo* on a 1.5T clinical platform. IVIM DWI allows for the estimation of a fast component of water diffusivity (f, D*, and fD*) related to renal perfusion and tubular excretion, in addition to measuring tissue diffusivity (D). A challenge of using IVIM DWI to measure renal function is the difficulty in decoupling vascular and tubular contributions, as the two are intimately linked through autoregulation to maintain homeostasis. Additional mechanistic insight may be possible by combining IVIM DWI data with quantitative T_2_* measurements using BOLD MRI, which reflects renal blood volume and tissue oxygenation. Studies have shown that increased T_2_* in the kidneys can result from increased hypoxia from higher tubular metabolism or increased blood volume.[23, 24] Combining IVIM DWI with T_2_* measurements allows corroboration of mutual information to explain changes in renal physiology with pharmacological/physiological intervention.

Repeatability measurements are critical for providing confidence in observed changes following experimental intervention, but are not routinely performed in pre-clinical imaging investigations. In this study, we first established the repeatability of the quantitative MRI biomarkers, with low CoVs for repeated measures demonstrating that the functional parameters did not suffer from instability over the imaging timecourse. Comparison of the perfusion-insensitive ADC and the IVIM parameter D showed good agreement both prior to and during challenge, suggesting that perfusion effects are mostly removed at b-values > 200 mm^-2^s. Although the validity of this assumption will vary with different tissues, this demonstrates the usefulness of the Bayesian fitting method used herein for removing this assumption and fitting the whole dataset, rather than the more common two-stage fitting of IVIM data. The use of clinical vendor surface coils used in parallel with volume coils provided sufficient signal and resolution to measure the MRI biomarkers with good repeatability. Unsurprisingly, measurement repeatability was poorer for the perfusion sensitive parameters compared with the perfusion-insensitive parameters and T_2_*.

We then proceeded to evaluate the effects of drugs known to modulate renal vascular and tubular function. Hydralazine is a well-characterised systemic vasodilator that relaxes vascular smooth muscle; the increased vascular fraction determined in the IVIM model, and reduction in T_2_*, is consistent with a hydralazine-induced increase in (deoxygenated) renal blood volume. Furthermore, the associated reduction in blood flow induces a compensatory increase in cardiac output to maintain blood pressure,[25] resulting in the observed increase in D* with net increased blood flow. To maintain fluid homeostasis, the kidney may also increase tubular transport, the resulting increase in oxygen consumption also contributing to the reduction in T_2_*. The decrease in the perfusion-insensitive ADC and D can be interpreted as a consequence of dehydration of the interstitial space.

Furosemide is a loop diuretic, inhibiting water reabsorption in the nephron by blocking the sodium-potassium-chloride co-transporter in the ascending limb of the loop of Henlé. The rapid and significant increase in T_2_* seen herein is consistent with previous reports and the known effects of furosemide on reducing renal blood volume and decreasing oxygen consumption.[14, 26, 27] A reduction in perfusion fraction in the renal cortex was also observed, suggesting a reactive decrease in vascular flow, and also consistent with a reduction in blood volume. Interestingly, the perfusion-insensitive ADC and D significantly increased, consistent with an increase in renal water content within the tubules due to diuretic effects. This contrasts with previous studies showing either no change, or a reduction in renal water diffusivity, in response to furosemide.[14]^,^[28]

Angiotensin II is a naturally-occurring hormone with a complex role within the renal renin-angiotensin system (RAS).[29] The hormone has a direct effect on the proximal tubules to increase Na^+^ reabsorption, and has a convoluted and variable effect on glomerular filtration and renal blood flow. Increases in systemic blood pressure will maintain renal perfusion pressure; however, constriction of the afferent and efferent glomerular arterioles can reduce renal blood flow. The effect on the efferent arteriolar resistance tends to increase glomerular capillary hydrostatic pressure and maintain glomerular filtration rate. In the present study, the initial reduction in T_2_* is consistent with an acute reduction in blood volume and tissue oxygenation, the subsequent recovery suggesting a reactive response to drug-induced vasoconstriction. A similar transient T_2_* response to angiotensin II has been reported in human kidney.[30] The DWI data revealed no significant changes, which supports the concept of an acute effective homeostatic response, and the absence of any vascular response in the IVIM parameters.

Animal welfare and ways of reducing animal usage is an important consideration for all research bioscientists; non-invasive and longitudinal imaging methods that incorporate repeatability measurements can reduce the number of animals by using each as its own control, harnessing statistical power through the use of paired statistical tests in small animal cohorts, and information that may relate better to that observed in clinical assessment of therapy efficacy. Clinical MRI scanners are being increasingly used for pre-clinical imaging studies. Compared to relatively expensive dedicated small-bore animal MRI systems, major vendors sell and distribute far more clinical scanners; consequences of this include the continuing development and availability of superior hardware and standardised pulse sequences on these platforms. Pre-clinical studies performed on clinical platforms also provide evidence supporting the clinical relevance of advanced diffusion modelling and data acquisition. Most clinical scanners operate at between 1.5 and 3 Tesla, and thus have lower signal-to-noise (SNR) levels than pre-clinical systems, with reduced image quality if conventional clinical imaging coils are used. This is exacerbated particularly when imaging the small fields-of-view necessary when using rodents, but with a high enough resolution to be able to acquire meaningful functional data. One approach for increasing SNR is to use small, dedicated receiver coils, such as the TMJ coil used herein, designed to fit closely to the object of interest, giving a better coupling between the object and coil with a corresponding increase in signal and thus improved image quality.[31] Here we have shown the sensitivity and stability of parallel imaging using standard vendor coils on a clinical 1.5T MR system for conducting rodent renal studies, and demonstrate the sensitivity of resulting DWI and BOLD MRI biomarkers to the effects of several vasomodulators. This experimental arrangement extends the available scope for performing pre-clinical studies with existing clinical hardware, and confers the advantages associated with increased access to clinical scanners for pre-clinical studies.

Some limitations in this study are clear. Firstly, due to the voxel size achievable on clinical 1.5T MR systems, we were limited in our ability to reliably draw smaller ROIs to interrogate regions within the kidney that may contain differential vascular/tubular components, and this was particularly true for DWI where signal is actively attenuated by the sensitising gradients. Hence, analysis was necessarily made over larger regions to ensure adequate image SNR. Secondly, the dynamic imaging protocol precluded the use of other complex invasive measurements to provide additional validation of our observations against other physiological measures over the experimental period.

**Generalisability/translation**

**19 Comment on whether, and how, the findings of this study are likely to translate to other species or systems, including any relevance to human biology.**

We have shown that IVIM DWI and T_2_* measurements are feasible on a 1.5T clinical system to monitor the acute effects of vascular and tubular modulating drugs on rat kidney function *in vivo*. Water diffusion in kidneys exhibits bi-exponential behaviour, with the fast diffusion component at low b-values reflecting both vascular and tubular flow. Multi-parametric MRI strategies combining IVIM DWI with T_2_* measurements allows mutual corroboration of pharmacological interventions. The administration of intravenous hydralazine, furosemide, or angiotensin showed differential effects on IVIM DWI and BOLD MRI biomarkers *in vivo*, and highlights the potential of these techniques to study the effects of drugs that modulate renal function in humans, so as to better understand their treatment effects in patients with renal dysfunction.

**Funding**

**20 List all funding sources (including grant number) and the role of the funder(s) in the study.**

We acknowledge the CR-UK support to the Cancer Imaging Centre at ICR and RMH in association with MRC & Department of Health C1060/A10334, C1060/A16464, C7809/A10342, NHS funding to the NIHR Biomedicine Research Centre and the Clinical Research Facility in Imaging.
